# miR-145 and miR-497 suppress TGF-β-induced epithelial–mesenchymal transition of non-small cell lung cancer by targeting MTDH

**DOI:** 10.1186/s12935-018-0601-4

**Published:** 2018-07-27

**Authors:** Qi Yin, Yang Han, Dongyi Zhu, Zhanxia Li, Shan Shan, Wenjing Jin, Qingchun Lu, Tao Ren

**Affiliations:** 1 Department of Respiratory Medicine, Shanghai East Hospital, Tongji University School of Medicine, Shanghai, 200120 China; 2 Department of Pathology, Shanghai Jiao Tong University Affiliated Sixth People’s Hospital, Shanghai, 200233 China; 3 Department of Intensive Care Unit, Shanghai East Hospital, Tongji University School of Medicine, Shanghai, 200120 China; 4 Department of Respiratory Medicine, Shanghai Jiao Tong University Affiliated Sixth People’s Hospital, Shanghai, 200233 China; 5 Department of Intensive Care Unit, Shanghai Pudong Hospital, Fudan University Pudong Medical Center, Shanghai, 201399 China

**Keywords:** Epithelial–mesenchymal transition, Non-small cell lung cancer, MTDH, miR-145, miR-497

## Abstract

**Background:**

MicroRNAs (miRNAs) have been reported to play crucial roles in multiple cancers including non-small cell lung cancer (NSCLC). Here, we investigated the role of miR-145 and miR-497 in TGF-β-induced epithelial–mesenchymal transition (EMT) process of NSCLC.

**Methods:**

We performed quantitative real time PCR (qRT-PCR) to detect the expression level of miR-145 and miR-497 in NSCLC cell lines. Then in the presence/absence of TGF-β, we transfected miRNA mimics or inhibitor into A549 and H1299 cells and investigated the role of miR-145 and miR-497 in cell migration and invasion using transwell and wound-healing assay. The regulation role of miR-145 and miR-497 on Metadherin (MTDH) was determined by luciferase assay. The expression level of MTDH and EMT markers E-cadherin and vimentin were detected on mRNA and protein level.

**Results:**

In our study, our results showed that miR-145 and miR-497 were downregulated in NSCLC cell lines. Overexpression of miR-145 and miR-497 inhibited TGF-β-induced EMT and suppressed cancer cell migration and invasion, while the opposite results were observed in cells transfected with miR-145 or miR-497 inhibitor. Moreover, the luciferase assay confirmed that miR-145 and miR-497 attenuated MTDH expression by directly binding 3′-UTR of MTDH mRNA and exert the tumor-suppression role.

**Conclusions:**

Overall, we demonstrated that miR-145 and miR-497 functioned as EMT-suppressor in NSCLC by targeting MTDH, provided new evidence that miR-145 and miR-497 as potential therapeutic targets.

## Background

The majority (79%) of non-small cell lung cancer (NSCLC) patients develop metastases from their primary tumor [1]. Metastasis is a complicated multistep involved in sequential and interrelated steps, including the epithelial–mesenchymal transition (EMT) process [2, 3]. In recent years, EMT has become the focus of attention as it plays a crucial role in cancer distal metastasis and invasion [4]. It is characterized by epithelial cells transform to mesenchymal-like cells, during which the cells lose cell–cell adhesion and acquire migratory and invasion properties [5]. The EMT process can be controlled by the canonical pathways such as Transforming growth factor-β (TGF-β)/Smad pathway and Wnt/β-catenin signaling [6]. For example, TGF-β induces EMT by repression of epithelial markers (such as E-cadherin) and gain of mesenchymal biomarkers (such as vimentin and N-cadherin) [7, 8]. Therefore, identification of the molecular mechanisms underlying EMT process is crucial for the expansion of our knowledge of tumor metastasis.

Metadherin (MTDH), also known as Astrocyte elevated gene-1 (AEG-1), was first cloned in primary human fetal astrocytes treated with TNF-α or infected with HIV-1 [9, 10]. MTDH is upregulated in multiple cancer tissues and cell lines, induce EMT via oncogenic signaling pathways including PI3 K/Akt, ERK, Wnt/β-catenin signaling, thus enhance tumor metastasis [11–14]. Elevated MTDH expression correlated with increased NSCLC proliferation and metastasis and predicted worse prognosis in patients [15–17]. These results demonstrated that MTDH may act as an important metastasis promoter in cancer development.

MicroRNAs (miRNAs) are a family of small non-coding RNAs of 18–22 nucleotides that negatively regulate gene expression by directly binding the 3′-UTR of mRNAs and cause mRNA degradation or translation suppression [18]. miRNAs play crucial roles in various of physiological and pathological processes including tumor metastasis and invasion [19]. A multitude of studies have revealed the important role of miRNAs in EMT process. For example, previous studies revealed that miR-145 and miR-497 suppressed cancer cell migration and invasion in gastric cancer and colorectal cancer cells, respectively [20, 21]. In breast carcinoma, miR-497 negatively regulates TGF-β-induced EMT by targeting Slug [22]. Another report showed that miR-145 was decreased in esophageal squamous cell carcinoma (ESCC) and its overexpression inhibited EMT in ESCC cells [23]. Moreover, lower miR-145 and miR-497 level was observed in NSCLC tissue [24, 25]. These results indicated that miR-145 or miR-497 may function as tumor suppressor in NSCLC.

In the present study, we investigated the role of miR-145 and miR-497 in EMT process. We provided evidence that miR-145 and miR-497 suppressed TGF-β-induced EMT, in NSCLC cell lines. Moreover, our study confirmed that oncogene MTDH was involved in this process and miR-145 and miR-497 modulated EMT by directly binding MTDH.

## Methods

### Cell culture

Three human NSCLC cell lines (A549, H1299 and H358) were obtained from the cell bank of Chinese Academy of Sciences (Shanghai, China), while the human bronchial epithelial cell line (HBE) was supplied by prof. Bailing Luo (Xiangya Hospital, Changsha, China). The cells were cultured in DMEM medium (Thermo Fisher Scientific, Waltham, MA, USA) containing 10% fetal bovine serum (Thermo) in a humidified incubator with 5% CO_2_.

### Cell transfection

miRNAs mimic, inhibitor and negative control were synthesized by GenePharma (Shanghai, China). The cells were transfected into H1299 and A549 cells by Lipofectamine 3000 (Thermo) according to the manufacturers’ protocol.

### RNA isolation and qRT-PCR

Total RNA was extracted from NSCLC cell lines using Trizol Reagent and reversed into cDNA using a RevertAid RT Transcription Kit (Thermo). The miRNA was isolated and reversed by Qiagen kit (Hilden, Germany). The PCR reaction was performed with SYBR Green Reagent (Thermo). The primers were designed as follows: MTDH-F, 5′-AAGCAGTGCAAAACAGTTCACG-3′ and MTDH-R, 5′-GCACCTTATCACGTTTACGCT-3′; GAPDH-F, 5′-GAGTCAACGGATTTGGTCGT-3′ and GAPDH-R, 5′-TTGATTTTGGAGGGATCTCG-3′. The forward primers for miR-145 and miR-497 were 5′-AGTCCAGTTTTCCCAGGAATCCCT-3′ and 5′-ACCAGCAGCACACTGTGGTTTGT-3′, while the reverse primer and U6 primers (internal control) were provided by Qiangen. The results were calculated by the 2^−ΔΔCt^ method.

### Western blot assay

The cells were lysed using RIPA buffer contained protease cocktail (Thermo) and PMSF. The homogenates were centrifuged and the supernatants were collected as total protein. Next, proteins were subjected to 10% SDS-PAGE gel electrophoresis and transferred to PVDF membranes. The membranes were blocked by 5% milk then incubated with primary antibodies against MTDH (1:10,000; Abcam, Cambridge, United Kingdom), E-cadherin (1:1000; Abcam), vimentin (1:3000; Abcam) and GAPDH (1:5000; Cell signaling Technology, Danvers, MA, USA) overnight. After incubating, blots were detected by secondary antibody and visualized by the ECL assay (Milliporesigma).

### Wound-healing assay

Cells were seeded in six-well plates and transfected so as to 100% confluence. Subsequently a p200 pipette-tip was used to create a scratch. Following this, the culture medium without FBS was added. Cells were imaged using an inverted microscope at 18 or 24 h after wounding.

### Transwell migration and invasion assay

The transwell migration and invasion assay were performed according to the manufactures’ protocols. Briefly, cells were suspended in 200 µl serum-free medium and seeded in the upper chamber (Corning, NY, USA). In the lower chamber medium with 20% FBS was added. After incubation for 24 h, cells on the surface of upper chamber were removed, and cells on the lower side of chamber were stained with crystal violet. The number of cell was counted in five random selected fields under a microscope, and the average cell number per field was determined.

### Dual luciferase assay

The bioinformatics tools, Starbase v2.0 and Targetscan were used to predict the miRNAs target. The MTDH 3′-UTR fragments with putative promotor region of miR-145 or miR-497 was synthesized and then inserted into the pMIR-REPORT luciferase vector (OBio Biology, Shanghai, China). For luciferase assay, cells were cultivated onto a 24-well plate and were co-transfected with pMIR-REPORT wt or mut plasmid, pRL-TK (Promega, Madison, WI, USA) and either miRNA mimics or negative controls. The luciferase activities were measured with the Dual-luciferase reporter assay system (Promega).

### Statistical analysis

Experimental data are presented as mean ± SD of three independent experiments. Student’s *t* test was used for statistical analysis. *P* < 0.05 was considered significant.

## Results

### miR-145 and miR-497 are downregulated in NSCLC cell lines

**Fig. 1:**
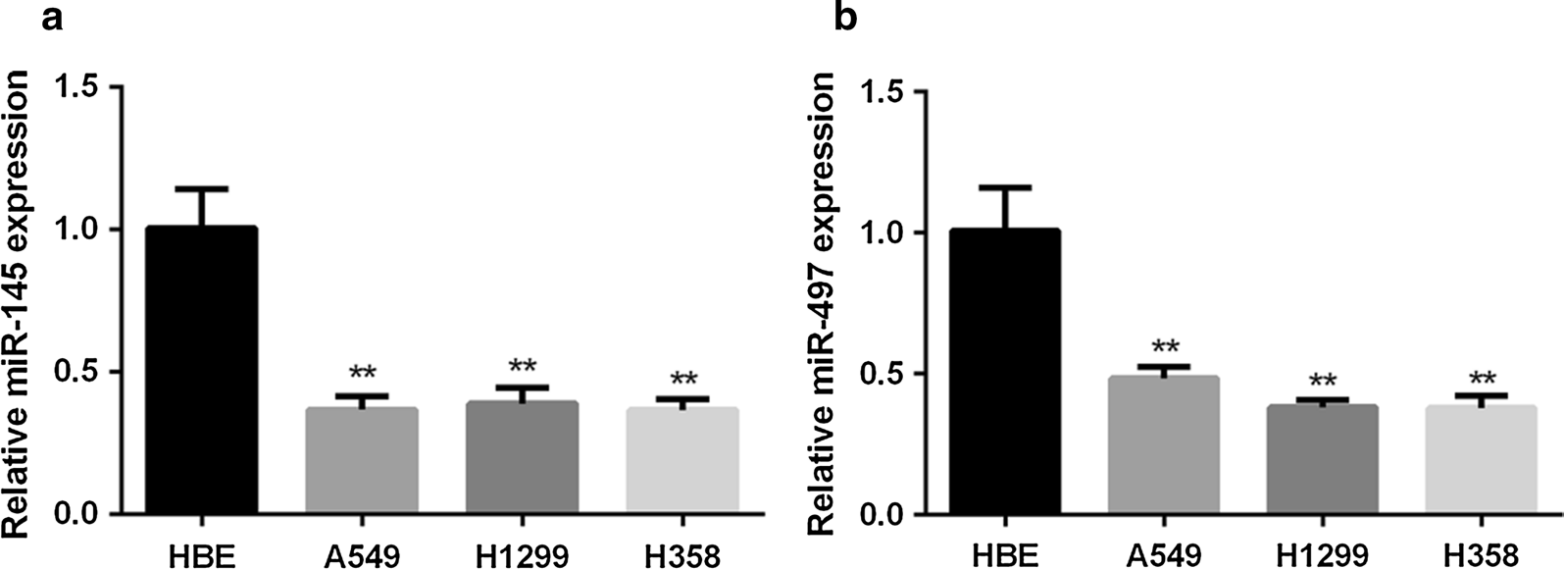
Expression of miR-145 and miR-497 in NSCLC cell lines. **a** Relative expression of miR-145 was detected by qRT-PCR in NSCLC cell lines and normal cell line HBE. **b** Relative expression of miR-497 in NSCLC cell lines and HBE cell line was evaluated by qRT-PCR. (**P < 0.05)

The expression levels of miR-145 and miR-497 in three NSCLC cell lines (A549, H1299 and H358) and HBE cell line were detected. Compared with HBE, miR-145 and miR-497 decreased in all the NSCLC cell lines (Fig. 1a, b). For further investigation, we chose epithelial cell line A549 and mesenchymal cell line H1299 in the following experiments.

### miR-145 and miR-497 inhibit TGF-β-induced migration and invasion

**Fig. 2:**
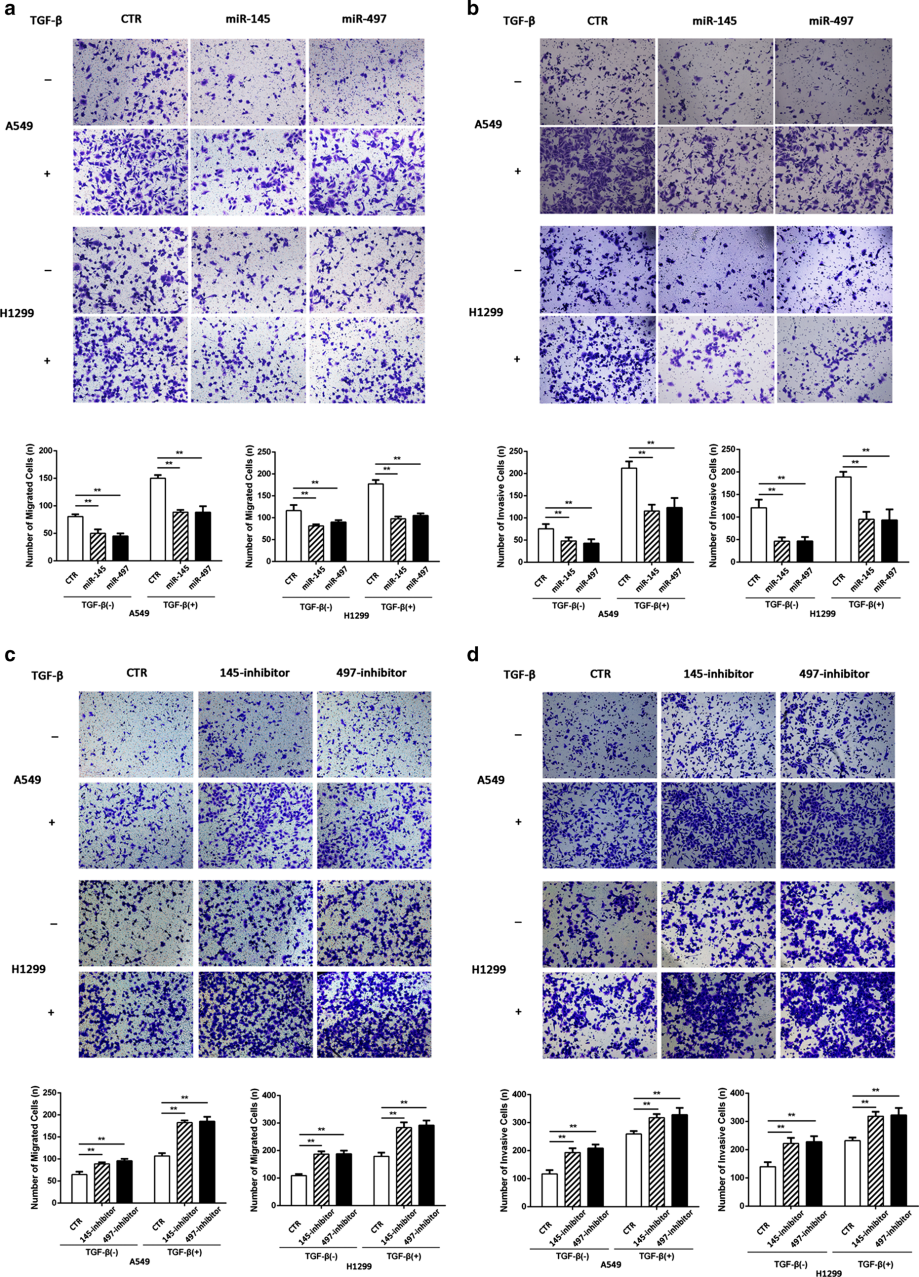
miR-145 and miR-497 in NSCLC cell migration and invasion. **a**, **b** After transfection of miR-145 or miR-497 mimic, cells were treated with or without TGF-β and transwell cell migration and invasion assays were performed. **c**, **d** Transfection with miR-145 and miR-497 inhibitor resulted in increased NSCLC cell migration and invasion rate in the presence/absence of TGF-β. Mimic NC or inhibitor NC (CTR) was used as control. (**P < 0.05)

**Fig. 3:**
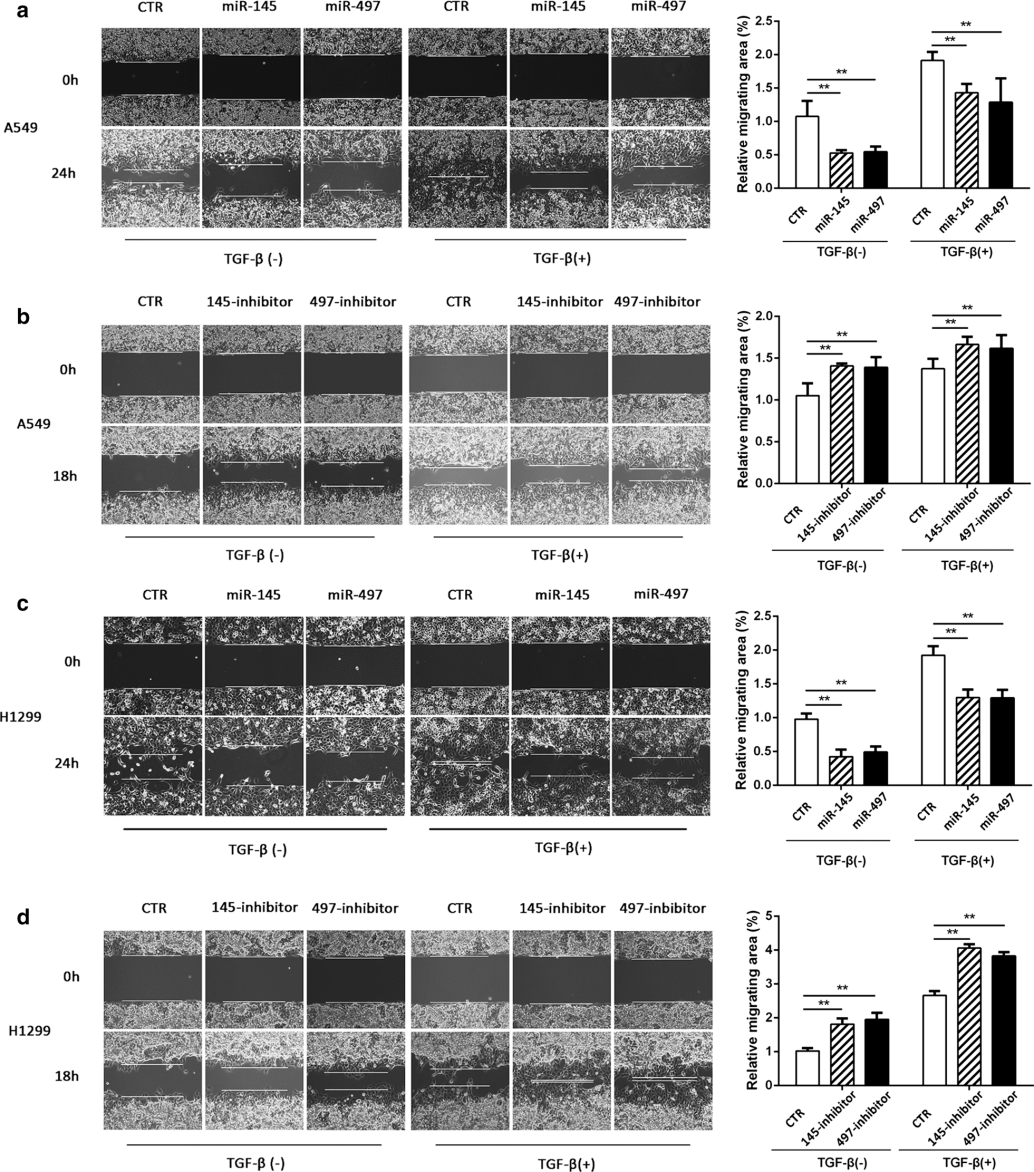
Wound-healing assay in NSCLC. **a**, **b** Relative migrating areas were evaluated in A549 cells transfected with miR-145/miR-497 mimic or inhibitor. **c**, **d** After transfection of miR-145/miR-497 mimic or inhibitor, cells were treated with/without TGF-β and the relative migrating areas of H1299 cell were detected.(**P < 0.05)

Since miR-145 and miR-497 were downregulated in NSCLC cell lines, we then investigated the role of miR-145 and miR-497 in TGF-β-induced cancer cell migration and invasion. Cells were transfected with miRNA mimic or inhibitor with lipo 3000. After 24 h, TGF-β was added and reached a final concentration of 10 ng/ml. The transwell experiments with or without matrigel revealed that either miR-145 or miR-197 upregulation suppressed NSCLC migration and invasion (Fig. 2a, b), while the opposite results were observed in cells transfected with inhibitor (Fig. 2c, d). Similar to above, the wound-healing assay showed that cell migration rate was suppressed by miR-145 and miR-497 (Fig. 3a–d). These data indicated that miR-145 and miR-497 could markedly inhibit the migratory and invasive capacity of NSCLC cell lines.

### MiR-145 and miR-497 suppress TGF-β-induced EMT

**Fig. 4:**
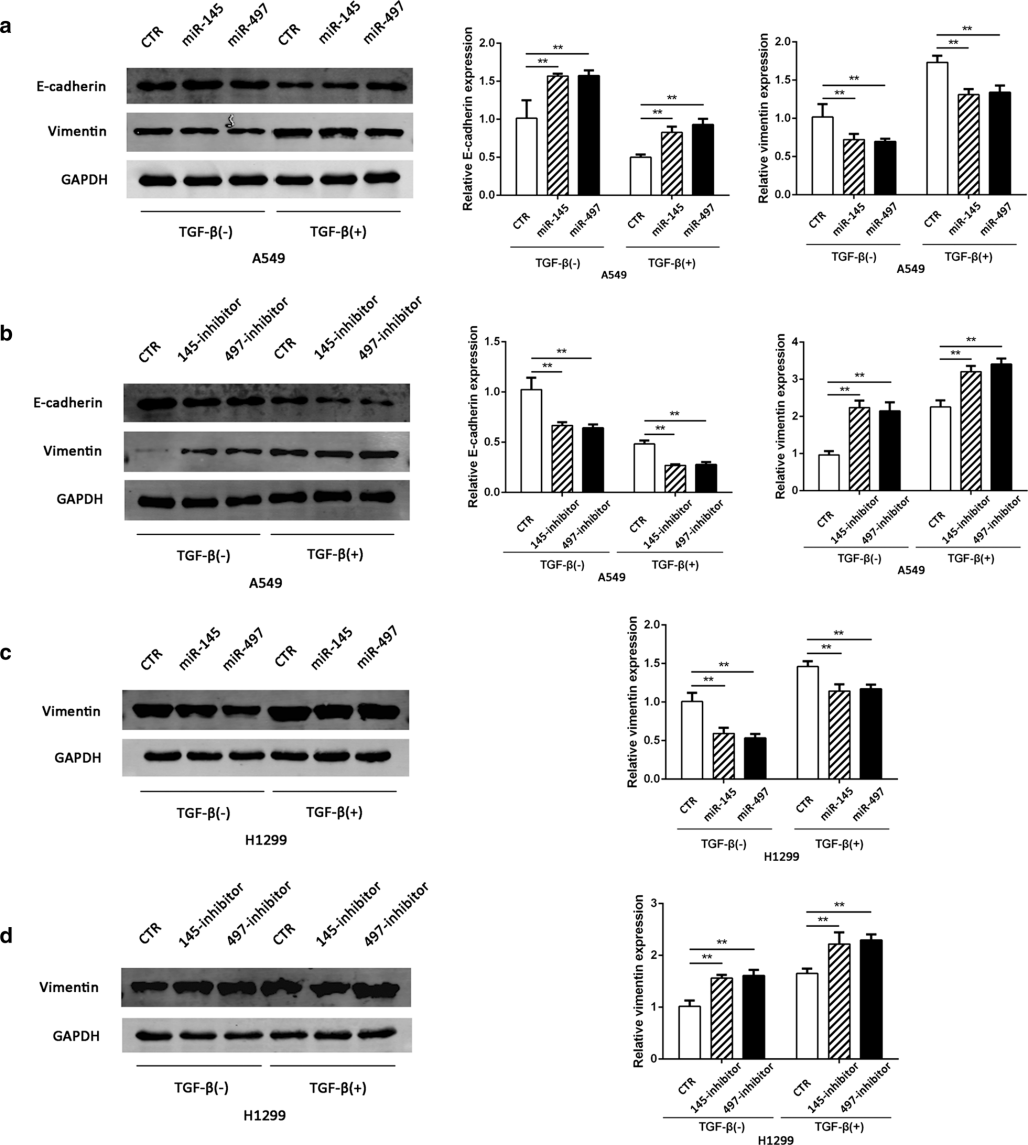
miR-145 and miR-497 inhibit EMT in NSCLC cells. **a**, **b** Western blot analysis for the expression of E-cadherin and vimentin in A549 cells treated with miR-145/miR-497 mimic or inhibitor. **c**, **d** The expression level of vimentin in H1299 cells transfected with miR-145/miR-497 mimic or inhibitor. (**P < 0.05)

Next we examined the potential effects of miR-145 and miR-497 on EMT features. For this, we detected the expression level of the EMT biomarkers, E-cadherin and vimentin. After incubation with 10 ng/ml TGF-β for 48 h, the epithelial marker E-cadherin decreased while mesenchymal maker vimentin increased in A549 cells. Overexpression of miR-145 and miR-497 correlated with upregulation of E-cadherin and downregulation of vimentin, and the opposite results were observed in A549 cells with decreased miR-145 or miR-497 (Fig. 4a, b). In H1299 cells the E-cadherin was undetectable due to its mesenchymal character, but the expression of vimentin had the similar results to that of A549 (Fig. 4c, d).

### MTDH is a direct target of miR-145 and miR-497

**Fig. 5:**
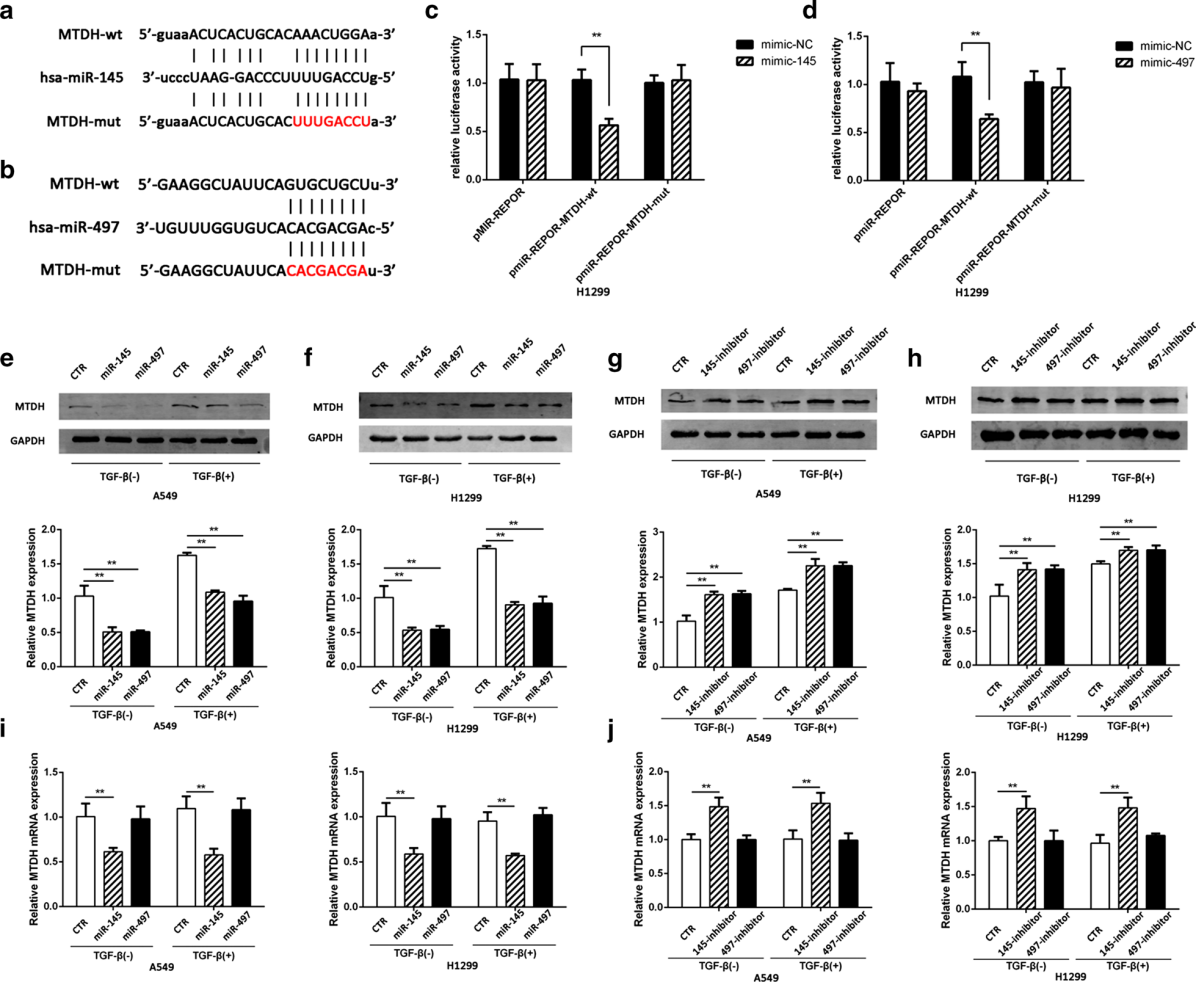
miR-145 and miR-497 directly target MTDH. **a**, **b** The potential binding sites of miR-145/miR-497 and MTDH were predicted by bioinformatics tools Starbase v2.0 and Targetscan. **c**, **d** Relative luciferase activity in cells co-transfected with wild-type/mutant plasmid and miR-145/miR-497 mimic were evaluated. **e**–**h** The expression levels of MTDH in A549 and H1299 cells transfected with miR-145/miR-497 mimic or inhibitor. **i**, **j** The mRNA levels of MTDH in A549 and H1299 cells transfected with miR-145/miR-497 mimic or inhibitor. (**P < 0.05)

To further investigate the mechanism of miR-145 and miR-497 in regulating NSCLC progression, we predicted the putative target of the two miRNAs and performed luciferase assay to verify the prediction (Fig. 5a, b). The results showed that both miR-145 and miR-497 significantly inhibited the luciferase reporter expression in H1299 cells transfected with MTDH 3′-UTR-wild type reporter but not those transfected with MTDH-mut or NC (Fig. 5c, d). Furthermore, the MTDH protein expression level was also inhibited by miR-145 and miR-497 mimic. On the contrary, inhibition of miR-145 and miR-497 increased MTDH expression on protein level (Fig. 5e–h). Despite the upregulation of MTDH protein, after stimulating with TGF-β, the level of MTDH mRNA was not changed. The mRNA level of MTDH was upregulated in cells transfected with miR-145 inhibitor and decreased in cells transfected with miR-145 mimic; however, MTDH mRNA level is not changed in cells incubating with miR-497 mimic or inhibitor (Fig. 5i, j).

## Discussion

It is widely known that EMT is a crucial process for the acquisition of cancer metastasis and invasion potential [26]. In the present study, we focused on two potential tumor-suppression miRNAs, miR-145 and miR-497, and found they were lower expressed in NSCLC cell lines compared with HBE. We also investigated the potential mechanism of the miRNAs and found that the overexpression of miR-145 and miR-497 inhibited TGF-β-induced EMT in NSCLC cell, and the miRNAs may exert its anti-cancer role by directly targeting an oncogene, MTDH. These results suggest that miR-145 and miR-497 may function as tumor-suppressor in NSCLC progression.

Recently, a series of miRNAs has been identified to modulate EMT process by modulating pivotal proteins [27]. For example, the miR-200 family plays an essential role in EMT suppression by modulating ZEB1 and ZEB2 [28]. Previous study showed that miR-145 and miR-203 inhibited TGF-β-induced EMT by modulating TGF-β/Smad3 pathway [4]. Zhang et al. reported that miR-497 was downregulated in colorectal cancer (CRC) cell and inhibited CRC cell EMT, migration and invasion [20]. To explore the role of miR-145 and miR-497 in EMT, we incubated the epithelial phenotype cell line A549 and mesenchymal phenotype cell line H1299 with TGF-β. After stimulating with TGF-β, the epithelial marker E-cadherin decreased while mesenchymal marker vimentin increased, in accordance with previous reports that TGF-β is an inducer of EMT [29]. In the presence/absence of TGF-β, transfection with miR-145 and miR-497 mimic resulted in the decreased percentage of migration and invasion cells. In A549 cells the expression level change of E-cadherin and vimentin was more obvious than H1299; this phenomenon may due to the mesenchymal character of H1299, which makes it a natural higher expression of vimentin. Based on these evidences, we provide the evidence that miR-145 and miR-497 are involved in TGF-β-induced EMT in NSCLC.

MTDH is a multifunctional oncogene and has been demonstrated to be activated in many malignancies, including gastric cancer, prostate cancer and glioma [13, 30, 31]. It has been reported that MTDH is a potential target of miR-145 and miR-497 [32–34]. Thus, we speculated that miR-145 and miR-497 may serve regulatory roles by suppressing the activation of MTDH in EMT. Here, we found that in NSCLC cell lines miR-145 and miR-497 could directly targeted MTDH, thus inhibited TGF-β-induced EMT. The results of qRT-PCR showed that overexpression of miR-145 correlated with MTDH mRNA downregulation. However, transfection with miR-497 mimic or inhibitor induced the upregulation or suppression of MTDH protein level but not the mRNA. These results suggest that miR-145 may degrades MTDH mRNA while miR-497 suppresses mRNA translation activity, provide evidence that downregulation of miR-145 and miR-497 may be essential for MTDH to exert its role in EMT.

Interestingly, TGF-β treatment resulted in the upregulation of MTDH protein but not the mRNA of MTDH. A recent study reported that in TGF-β treated malignant glioma, MTDH protein was increased via Smad2/3 phosphorylation [30]. In activated TGF-β/Smad signaling pathway, the p-Smad2 and p-Smad3 form oligomeric complexes with Smad4, translocated into the nucleus and regulated genes transcription [35, 36]. However, in our study, we demonstrated that TGF-β treatment perform functions on translational regulation of MTDH rather than transcriptional repression. This result suggested that other potential pathways may exist between TGF-β/Smad and MTDH, such as miRNA/mRNA regulatory mechanism. Based on our present study, we speculate that miR-145 and miR-497 may participate in this regulatory network.

## Conclusions

In conclusion, our results suggested that miR-145 and miR-497 played crucial roles in TGF-β-induced EMT by regulating MTDH in NSCLC cell. Our understanding of the miRNA-based mechanism might contribute to discover novel therapeutic target for NSCLC.

## Abbreviations

EMT: epithelial-to-mesenchymal transition
NSCLC: non-small cell lung cancer
TGF-β: transforming growth factor-β
ECL: electrochemiluminescence
PMSF: phenylmethanesulfonyl fluoride
PCR: polymerase chain reaction
FBS: fetal bovine serum
MTDH: metadherin
HBE: human bronchial epithelial cell line
